# Extracellular Vesicles: Delivery Vehicles of Myokines

**DOI:** 10.3389/fphys.2019.00522

**Published:** 2019-05-07

**Authors:** Eleonora Trovato, Valentina Di Felice, Rosario Barone

**Affiliations:** ^1^Department of Biomedicine, Neurosciences and Advanced Diagnostic (BIND), Human Anatomy and Histology Institute, University of Palermo, Palermo, Italy; ^2^Innovation and Biotechnology for Health and Exercise (iBioTHEx), Palermo, Italy; ^3^Euro-Mediterranean Institute of Science and Technology (IEMEST), Palermo, Italy

**Keywords:** exercise, muscle cells, exocytosis, extracellular bodies, exosomes

## Abstract

Movement and regular physical activity are two important factors that help the human body prevent, reduce and treat different chronic diseases such as obesity, type 2 diabetes, heart diseases, hypertension, sarcopenia, cachexia and cancer. During exercise, several tissues release molecules into the blood stream, and are able to mediate beneficial effects throughout the whole body. In particular, contracting skeletal muscle cells have the capacity to communicate with other organs through the release of humoral factors that play an important role in the mechanisms of adaptation to physical exercise. These muscle-derived factors, today recognized as myokines, act as endocrine and paracrine hormones. Moreover, exercise may stimulate the release of small membranous vesicles into circulation, whose composition is influenced by the same exercise. Combining the two hypotheses, these molecules related to exercise, named exer-kines, might be secreted from muscle cells inside small vesicles (nanovesicles). These could act as messengers in tissue cross talk during physical exercise. Thanks to their ability to deliver useful molecules (such as proteins and miRNA) in both physiological and pathological conditions, extracellular vesicles can be thought of as promising candidates for potential therapeutic and diagnostic applications for several diseases.

## Introduction

Skeletal muscle is the largest organ of our body, responsible for our posture and our movement. It is equivalent to 2/5 of the whole body weight and it is responsible for more than 3/4 of the total human metabolism. It is mainly composed of proteins and a fine balance between protein synthesis and protein degradation regulates its mass. An unbalance in one of these two processes can lead to the establishment and progression of pathological conditions (Bowen et al., 2015) such as sarcopenia (slow and normal loss of muscle due to aging, in absence of other diseases) and cachexia (multifactorial syndrome, characterized by a severe and involuntary loss of muscle mass, with or without loss of fat mass) (Fearon et al., 2011).

A sedentary lifestyle, physical inactivity and malnutrition (reduction or hyper-caloric intake), are among the causes that emphasize the accumulation of visceral fat. Therefore, a lifestyle based on a greater physical activity and a lower energy intake, helps to decrease visceral fat mass content (Miyatake et al., 2002; Shojaee-Moradie et al., 2007), inflammation (Petersen and Pedersen, 2005; Mathur and Pedersen, 2008; Nilsson et al., 2019) and the risk of several chronic diseases such as obesity (Roh and So, 2017), type 2 diabetes (Hu et al., 1999) and cancer (Hojman et al., 2011; Barone et al., 2016).

Muscle fiber is the principal unit of skeletal muscle and it is able to shorten its length because of nervous stimulation. Its development requires the involvement of several proteins and it is promoted by the differentiation and fusion of muscle cell progenitors into myotubes (Mauro, 1961). During physical activity, these cells are subjected to energetic (metabolic) and mechanical (contractile) stimuli that improve metabolic health of skeletal muscle and promote the release of specific molecules (called myokines), that can alter the function of other tissues (Stanford and Goodyear, 2018).

Recently, physical activity has also been associated with the release of extracellular vesicles (EVs) into the circulation (Fruhbeis et al., 2015). These are nano-sized vesicles that appear to be involved into cell-to-cell communication and may probably bring cytokines to distal organs, such as the heart (Bei et al., 2017). Communication is an essential process in multicellular organisms, both in physiological and pathological conditions and it is actuated by the exchange of information through different mechanisms: direct contact between cells (Cartwright and Arnold, 1980; Kalimi and Lo, 1988; Franke, 2009), secretion of soluble factors (Sicard, 1986; Lukacs et al., 1995) or interaction ligand-receptor (Qi et al., 2001). They are spherical organelles (originating from intracellular lipid compartments and released into the extracellular space and the systemic circulation) that have been discovered as new protagonists of intercellular communication (Pap et al., 2009; Zomer et al., 2010; Desrochers et al., 2016; Verweij et al., 2019).

Extracellular vesicles were first considered as cell waste products, but several studies revealed that they can transfer signaling molecules among cells in an autocrine, paracrine or endocrine manner (Lobb et al., 2015). They play a crucial role in regulating physiological processes (Schweitzer, 1973; Ratajczak et al., 2006; Kurachi et al., 2016; Niu et al., 2016; Bidarimath et al., 2017), inducing local and systemic changes that can develop, in some cases, into the progression of some diseases like cancer (Becker et al., 2016; Ohyashiki et al., 2018), neurodegenerative diseases (Vella et al., 2007; Danzer et al., 2012; Guix et al., 2018) and viral infections (Jaworski et al., 2014; Kalamvoki et al., 2014).

Interest in EVs has grown exponentially in the last 30 years, in part because most of the cells are able to secrete them. Skeletal muscle, the organ most represented in our organism, with its mechanical and secretory activities (Pedersen, 2013) may be responsible for the release of most of the circulating EVs during exercise. In fact, our research group has recently demonstrated that a single bout of exercise induces an increase in the release of EVs in the blood of healthy mice (Barone et al., 2016). While another paper described how an event of injury in response to exercise alters the composition of circulating EVs such as the content of some micro-RNAs (mi-Rs) (Guescini et al., 2015; Lovett et al., 2018).

## Myokines and the Contracting Muscle

Physical activity is known to exert beneficial effects on the prevention of chronic diseases (Zheng et al., 2006;Mikus et al., 2010; Pedersen and Saltin, 2015), this may be due to the release of contraction-regulated molecules (cytokines and myokines) that play a crucial role in the communication between muscle and other tissues (such as adipose tissue, liver, and pancreatic cells) (Ahima and Park, 2015). Despite the term “myokine” is generally referred to any secreted protein synthesized by the skeletal muscle tissue, it should be effectively used to describe only those proteins secreted by muscle cells. In fact, the skeletal muscle also comprise fibrous connective tissue and endothelial and nerve cells.

**Table 1:** Principal myokines and exerkines released and regulated during physical activity.

Contracting Exerkines	Levels of myokine	Reference
FGF-21	Increase	Cuevas-Ramos et al., 2012; Kim and Song, 2017
IL-6	Increase	Papanicolaou et al., 1996
IL-8	Increase	Mucci et al., 2000; Covington et al., 2016
IL-15	Increase	Nielsen et al., 2007; Hingorjo et al., 2018
Irisin	Increase	Kraemer et al., 2014
BDNF	Increase	Gomez-Pinilla et al., 2002; Fortunato et al., 2018
ANGPTL4	Increase	Norheim et al., 2014
LIF	Increase	Broholm et al., 2011
FSTL-1	Increase	Xi et al., 2016
VEGF	Increase	Gustafsson et al., 2001; Gomes et al., 2017
Myostatin	Decrease	Hittel et al., 2010

Several myokines are synthesized and secreted during contraction (Pourteymour et al., 2017) like Fibroblast Growth Factor 21 (FGF-21) (Kim and Song, 2017), Interleukin-6 (IL-6) (Brown et al., 2018), Interleukin-8 (IL-8) (Covington et al., 2016), Interleukin-15 (IL-15) (Hingorjo et al., 2018), Leukemia Inhibitory Factor (LIF) (Broholm et al., 2011), irisin (Lu et al., 2016), Myostatin (Hjorth et al., 2016), Angiopoietin-like 4 (ANGPTL4) (Norheim et al., 2014), Brain-Derived Neurotrophic Factor (BDNF) (Fortunato et al., 2018), Follistatin-like 1 (FSTL1) (Xi et al., 2016) and Vascular Endothelial Growth Factor (VEGF) (Gomes et al., 2017). Their expression in skeletal muscle is generally very low, but the levels of some of these myokines increase considerably during muscle contraction (FGF-21, IL-6, IL-15, irisin and BDNF among others) (Table 1). Moreover, the use of different exercise-based protocols (aerobic or resistance exercise training) can affect their secretion (Guescini et al., 2015; Schild et al., 2016; Abd El-Kader and Al-Shreef, 2018; Brown et al., 2018).

FGF-21 is an endocrine hormone belonging to the family of fibroblast growth factors (FGFs), which plays an important role in response to liver starvation, in lipolysis and glucose uptake in adipose tissues and skeletal muscle. It enhances the utilization of energy substrates (fatty acids, ketones and glucose) and meddles with energy consuming processes (lipogenesis and growth). Moreover, under certain conditions (such as cold), it can stimulate the activation of brown adipocytes and promote adaptive thermogenesis (Wu et al., 2013). It is also involved in mechanisms related to physical activity; in fact, its levels increase in acute exercise (in both human and mice), enhancing the phosphorylation of protein kinase B (Akt) and the translocation of Glucose Transporter Type 4 (GLUT4) to the muscle cell membrane, with consequent glucose uptake (Tanimura et al., 2016). Some results are controversial. For example, Cuevas-Ramos et al. (2012) proved that its serum levels increase only after 2 weeks of daily physical activity and no change was observed after a single bout of exercise. Chronic exercise and FGF-21 also induces the expression of Peroxisome proliferator-activated receptor Gamma Coactivator -1α (PGC-1α), a transcriptional protein factor that interacts with various DNA-binding proteins resulting in increased gluco-neogenesis, fatty acid oxidation, ketogenesis and mitochondrial biogenesis (Barone et al., 2016, 2017).

Interleukins are another important class of molecules released during exercise, especially IL-6, IL-8 and IL-15 (Ostrowski et al., 1999; Peake et al., 2015). They are small molecules belonging to the class of cytokines and constitute one of the most important communication systems among cells, defending the body (Nelson and Summer, 1998).

IL-6 is a pleiotropic cytokine, able to work as a pro- and anti-inflammatory molecule (Petersen and Pedersen, 2006; Yao et al., 2014), cardiovascular risk factor and regulator of lipid metabolism (Bao et al., 2015). It also acts as a myokine and its concentration increases up to 100-fold in response to muscle contraction (Agarwal et al., 2017), mediating anti-inflammatory responses and metabolic adaptations. It may reduce the incidence of cardiovascular diseases through lipid and glucose metabolism and the suppression of pro-inflammatory cytokines. During exercise, it acts both locally (inside the muscle) and peripherally (in several organs, such as white adipose tissue WAT) acting as a hormone. In WAT, it affects adipose tissue metabolism and lipolysis, with the release in the circulation and oxidation of fatty acids (Knudsen et al., 2017) while in skeletal muscle, it activates 5′-Adenosine Monophosphate-activated Protein (AMP) and/or Phosphatidylinositol 3-kinase (PI3 kinase) to increase glucose uptake and fat oxidation (Pedersen, 2009).

IL-15 is one of the most abundantly expressed cytokines in human muscle, involved in the regulation of adiposity, muscle mass, exercise capacity and mitochondrial activity in muscle cells (Thornton et al., 2016). Historically studied as an activator of Natural Killer (NK) cells (with anti-tumorigenic and anti-inflammatory properties) (Gosselin et al., 1999), its levels strongly increase after resistance exercise, promoting muscle growth (Nielsen et al., 2007). Similar to IL-6, its activity is strongly correlated to the AMP-protein kinase (AMPK), a central regulator of metabolism; indeed, it was shown that lacking muscle AMPK reduces serum IL-15, causing the acceleration of skin aging (Crane et al., 2015). IL-15 has also anti-tumorigenic effects; in fact, Carbo et al. (2000) showed that, in tumorigenic rats treated with IL-15, it partly inhibits skeletal muscle wasting protein rates (eight-fold) to values even lower than those observed in non-tumor-bearing animals.

Irisin is a secreted myokine that originates from the cleavage of its precursor fibronectin type III domain-containing protein 5 (FNDC5) (Huh et al., 2012). Firstly known as a molecule responsible for the browning of WAT (Giralt and Villarroya, 2013), it was also shown that its levels strongly increase after resistance exercise (Miyamoto-Mikami et al., 2015; Zhao et al., 2017), but not in aerobic training (Kim et al., 2016). Moreover, it was also recently proposed as a novel marker for patients with cardiac cachexia (Kalkan et al., 2018). Even if it was shown that it can mediate those beneficial effects that follow exercise (inducing the expression of pro-myogenic genes in myotubes), its role in physical activity still remain controversial. In fact, on one hand, Blizzard Leblanc et al. (2017) shown a significant increase in irisin plasma levels after an acute bout of aerobic exercise, also associated with the improvement in insulin sensitivity. These data were also confirmed by Nygaard et al. (2015), which further showed that single sessions of intense endurance exercise and heavy strength training led to transient increase of plasma concentration, without an increase in FNDC5 expression (Pang et al., 2018). However, on the other hand, a pivot study in hemo-dyalised patients proved that there was no correlation between intense intra-dialytic strength exercise and the increase of circulating irisin (Esgalhado et al., 2018). Also, the research group of Biniaminov et al. (2018) investigated the association among resting irisin concentrations, regular physical activity and physical fitness in serum of healthy humans; they found that nor physical activity level, neither fitness status were related to resting irisin concentrations in healthy humans.

Considering its potential beneficial effects during training, Reza et al. (2017) investigated its capability to act as an exercise mimetic. They injected wild-type mice with irisin and observed an increase in body weight, skeletal muscle mass and muscle strength, suggesting that irisin induces fiber hypertrophy and enhances regeneration of injured muscle cells, through the activation of satellite cells and protein synthesis.

Overall, the way irisin works is still largely unknown. However, it may be that it exerts its activity in a way similar to that of interleukins, through AMP kinase.

Another important myokine released during physical exercise is the BDNF (Gomez-Pinilla et al., 2002), a type of neurotrophic molecule mainly involved in memory and cognitive development (Heldt et al., 2007). This molecule is the product of the proteolytic cleavage of its precursor protein (proBDNF) (Koshimizu et al., 2009). BDNF and proBDNF often have opposing actions; in fact, while BDNF promotes synaptic long-term potentiation and stress resistance, proBDNF enhances longterm depression (Teng et al., 2005).

Brain-derived neurotrophic factor has neurobiological and metabolic effects, regulating the survival (Hofer and Barde, 1988), growth (Kalcheim and Gendreau, 1988) and maintenance of neurons (Griesbeck et al., 1995). Its association with physical activity is already known (Almeida et al., 2016; Church et al., 2016); in fact, its expression not only increases after exercise (Park and Kwak, 2017) but also depends on the intensity of exercise (Jeon and Ha, 2017). Previous studies demonstrated that its mRNA and protein expression levels increase in human skeletal muscle after exercise without any release into the circulation (Matthews et al., 2009). Indeed, it acts as a contraction-inducible protein that enhances fatty acid oxidation through the activation of AMPK in the skeletal muscle. Thus, it does not act in a hormone like manner, but as an autocrine and/or paracrine molecule within the skeletal muscle tissue (Matthews et al., 2015).

**Table 2:** Types of extracellular vesicles and most common markers.

	Apoptotic Bodies	Microvesicles-Ectosomes	Exosomes (small-medium-large)
Size	>800 nm	0.1 nm–1 μm	30–150 nm
Biogenesis	Apoptosis	Outward budding of plasma membrane	Endocytosis
Common Markers	Chemokine (C-X3-C motif) ligand 1 X3CL1 Intercellular adhesion molecule 3 (ICAM-3)	β1 integrins; Selectins CD40 Matrix metalloproteinase (MMP)	Tetraspanins (CD63; CD9; CD81) Hsps (Hsp70; Hsp90) Tumor susceptibility gene 101 (TSG101) Annexin V-VI Metallopeptidase domain 10 (ADAM10) Alix
Reference	Groves and Ellwood, 1985; Catchpoole and Stewart, 1995	Surman et al., 2017	Kowal et al., 2016

## Extracellular Vesicles: Origins and Functions

First characterized in 1967 in hematopoietic cells (Wolf, 1967), EVs are small particles composed of a lipid bi-layer containing multiple molecules derived from the cytosol and from the cellular membrane of the donor cell (Akers et al., 2013). They constitute a heterogeneous population that differs in cellular origin, size, morphology, antigenic composition and functional properties (Table 2). Isolated from different body fluids (including blood, urine, saliva, breast milk, amniotic fluid, ascites, cerebrospinal fluid, bile and seminal fluid) (Colombo et al., 2014), these vesicles are involved in the communication of both prokaryotes and eukaryotes. Despite the fact that their name originally referred to their size (apoptotic bodies > 800 nm, microparticles in a range of 0.1–1 μm and exosomes with diameter of 40–150 nm), their tissue of origin (prostasomes, oncosomes) and their function or their presence outside the cells (exosomes), one of the last classifications of EVs, focused on their biogenesis pathways (Kalra et al., 2016). However, there is still no consensus on specific EV subtypes markers, such as endosome-origin “exosomes” and plasma membrane-derived “ectosomes” (microparticles/microvesicles); therefore, assigning an EV to a particular biogenesis pathway remains difficult unless the EV is caught during its release in live imaging techniques. Hence, in order to classify a particular vesicle to a specific EV subtype, MISEV 2018 suggested considering several parameters such as: size [“small EVs” (sEVs) and “medium/large EVs” (m/l-EVs)], density (low, middle, high), biochemical composition (CD63+, CD81+, Annexin A5), description of their tissue of origin (prostasomes, oncosomes), their function or their presence outside the cells, or their biogenesis pathway (Théry et al., 2018).

Extracellular vesicles obtained from differential ultracentrifugation have been classified into: large EVs, pelleted at low speed; medium-sized EVs, pelleted at intermediate speed and small EVs (sEVs), pelleted at high speed. The latter were further subdivided into four sub-categories: (1) sEVs rich in tetraspanines CD63, CD9 and CD81 and endosomal markers (better known as exosomes); (2) sEVs without CD63 and CD81, but rich in CD9; (3) sEVs without CD63/CD9/CD81; (4) sEVs rich in extracellular matrix or serum factors. The last two-listed sEV are not associated with exosomes (Kowal et al., 2016).

Their molecular composition reflects the specific functions of the cells from which they originate and, for this reason, their cargo is defined as cell-type specific. They generally carry several molecules, such as proteins, nucleic acids (largely represented by mRNA and miRNA) and lipids, and their protein composition is similar to that of the plasma membrane and of the endocytotic and subcellular compartments of the budding cell (Gutierrez-Vazquez et al., 2013). In particular, exosome protein content is mainly divided in four categories: transmembrane or lipid bound extracellular proteins (tetraspaninins like CD9, CD81, CD63), cytosolic proteins normally involved in their biogenesis (RAB proteins, Hsp70, Hsp90), intracellular proteins unique of cellular organelles and typically absent in exosomes (calnexin, Golgi and ER proteins) and extracellular proteins, such as acetylcholinesterase (ACHE). When characterizing these particles, at least one protein of each category should be identified (Lotvall et al., 2014). Therefore, membrane and cytoskeletal proteins, lysosomal markers enzymes, death receptors (FasL, TRAIL), cytokines, HLA class I and II proteins, and some HSPs can be part of these vesicles. As well as their molecular content, even their functions are closely related to their cellular origin, being involved in several mechanisms such as immune response and inflammation (Zhang et al., 2014).

Despite the fact that the lipid composition of these nanovesicles is still not well known, we know that EVs are mainly composed of sphyngolipids, phosphatidyl serine, cholesterol (possibly involved in exosomes release), saturated fatty acids and ceramide (Pfrieger and Vitale, 2018).

The mechanisms underlying the biogenesis of EVs are different among various types of vesicles. Apoptotic bodies (>800 nm) are particles that cells produce during the apoptotic process. During programmed cell death, membrane protrusion known as apoptopodia (Atkin-Smith et al., 2015) release (in the extracellular space) vesicles (apoptotic bodies) resulting from the fragmentation of the apoptotic cell (Coleman et al., 2001). Ectosomes (0.1–1 μm), also known as shedding vesicles or microparticles, originate from plasma membrane through an outward budding of the membrane; in this process, the increase of intracellular Ca^2+^, induced by an external signal, causes changes in lipid distribution and membrane blebbing, through the alteration of the enzymatic activity of flippases, translocases and scramblases (Willms et al., 2018). This increment of internal Ca^2+^ enhances the activation of cytosolic proteases (such as calpain and gelsolin), which re-organize the cytoskeleton (through the deconstruction of the actin cytoskeletal protein network) and causing plasma membrane protrusion with consequent detachment of these vesicles (Turturici et al., 2014; Cocucci and Meldolesi, 2015).

**FIGURE 1:**
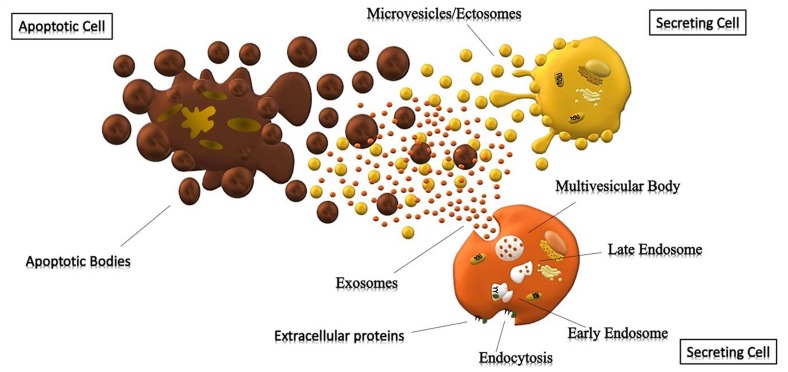
Cells releasing extracellular vesicles. Representation of different extracellular vesicles. Brown particles represent Apoptotic Bodies (>500 nm), forming during the apoptotic process; while the yellow and orange vesicles represent Microparticles (>150 nm) and Exosomes (30–150 nm) generated respectively by outward budding of the membrane and endocytosis.

Exosomes (<150 nm) are the most studied small vesicles, especially because of their small size and internal content that reflects that of the cell of origin. They are mostly defined by their size and their protein content, despite the fact that in literature the term “exosome” is improperly used to refer to small EVs (Wang et al., 2017). Their biogenesis is a well-organized process, mainly characterized by exocytosis through an active involvement of the membrane. The process starts with the invagination of the plasma membrane and the development of the early endosome (EE), a membrane bounded compartment within the cell. Subsequently, the inner budding of the membrane of the early endosome replaces the already existing endosomal luminal space with small intraluminal vesicles (ILVs) and forms a body called Multi-Vescicular Body (MVB) or late endosome (LE). The latter is filled with proteins, lipids, and cytoplasm specifically sorted (Gutierrez-Vazquez et al., 2013; Hessvik and Llorente, 2018). At this point vesicles within the MVB can undergo three different fates: merge with the lysosomes and be degraded in their protein content; constitute a momentary deposit compartment or merge with the plasma membrane, releasing its intraluminar vesicles in the extracellular space as exosomes (Figure 1) (Caruso Bavisotto et al., 2017; Sutaria et al., 2017). The development of ILVs and MVBs is a process that requires the participation of the Endosomal Sorting Complex Required for Transport (ESCRT), a complex composed by almost 30 proteins assembled into 4 components: ESCRT 0, ESCRT I, ESCRT II and ESCRT III. ESCRT 0 is involved in the recognition and the sequestration of ubiquitinated transmembrane proteins into the endosomal membrane; ESCRT II and I are responsible for the membrane deformation into buds with specific cargo; the last complex is implicated into the detachment of the formed vesicle. Even though the mechanism involved in exosomes secretion is still not well understood, it is likely that the increase of internal Ca^2+^, followed by a cytoskeleton remodeling, is involved in their release. Once in the extracellular space, these vesicles can be either internalized by the receiving cell through endocytosis processes, or they act as transmembrane signals by binding receptors on the plasma membrane and activate specific cellular pathways (Feng et al., 2010; Mulcahy et al., 2014). It was also thought that their uptake can be due by a “passive endocytosis” which occurs during the natural recycling of the plasma membrane and could passively take up exosomes attached to the surface of a cell.

**Table 3:** Most common extracellular vesicles isolation and characterization methods.

Isolation	References	Characterization	References
Ultracentrifugation	Thery et al., 2006	Electron Microscopy: TEM; SEM; cryo-EM	Escola et al., 1998; Poliakov et al., 2009; Sokolova et al., 2011
Density gradient Ultracentrifugation	Zhang et al., 2014	Dynamic Light Scattering (DLS); Nanoparticle Tracking Analysis (NTA); Tunable Resistive Pulse Sensing (TRPS)	Kesimer and Gupta, 2015 Gercel-Taylor et al., 2012 Maas et al., 2014
Size Exclusion Chromatography (SEC)	Boing et al., 2014	Biochemical analysis (Western Blot)	Théry et al., 2018
Immunological separation: Magnetic beads; ELISA exoTEST	Logozzi et al., 2009; Pedersen et al., 2017	Flow cytometry	Pospichalova et al., 2015
Polymer-based precipitation	Brown and Yin, 2017	Omics analysis: Proteomic; Lipidomic	Schey et al., 2015; Haraszti et al., 2016
Flow-field-flow fractionation	Sitar et al., 2015		
Commercial Kits ExoQuick Total Exosomes Isolation (TEI) Exo-Spin (ExoS)	Ding et al., 2018		

Currently, the characterization of these vesicles is a combination of several methods that include microscopy (TEM, SEM, CrioTEM), Western Blot, Flow Cytometry, Nanoparticle Tracking Analysis (NTA), Tunable Resistive Pulse Sensing (TRPS), Dynamic Light Scattering (DLS) and immuno-histochemical analysis of specific EVs markers, used to describe their morphology, biochemical composition and the receptors localized on these vesicles (Szatanek et al., 2015) (Table 3).

However, one of the main issue when purifying these particles is that, currently, there is no consensus for a unique standard isolation protocol. Ideally, the method used for their isolation should be simple, fast and inexpensive.

Overall, there are three main methodologies used for their isolation: differential centrifugation/ultracentrifugation with/without a sucrose gradient/cushion, adsorption to magnetic/non-magnetic micro-beads and size exclusion chromatography. Each has its own advantages and the choice of one method rather than another can result in different EV subpopulations with different properties (Konoshenko et al., 2018). Hence, one important challenge is the absence of a unique method able to minimize co-isolating protein aggregates and other membranous particles from a pure sample of EVs. Currently, the gold standard for pure exosome preparation is differential ultracentrifugation coupled with sucrose/iodixanol density gradient. Van Deun et al. (2014) isolated EVs with 4 commonly used methods (ultracentrifugation, density gradient, exo-kit and total exosome isolation) for the evaluation of yield, size, morphology, protein and RNA content of exosome. They demonstrated that density gradient ultracentrifugation gave the purest exosome preparations, while the other three techniques also co-isolate contaminating factors. Similarly, Skottvoll et al. (2019) evaluated the performance of different isolation methods based on differential ultracentrifugation and a commercial isolation kit (total exosome isolation reagent). These results shown that the two isolation methods had similar performance with only some differences based on the origin of the cell. In another study, the results published by Gamez-Valero et al. (2016) suggested that size-exclusion chromatography (SEC) is capable of eliminating most of the abundant proteins contained in a body fluid, also maintaining the EVs vesicular structure and conformation, thus making this procedure ideal for biomarker discovery as well as for therapeutic applications. Benedikter et al. (2017) also confirmed these data, demonstrating that ultrafiltration followed by size exclusion chromatography (UF-SEC), provides well-concentrated EVs for proteomic and functional analysis. Indeed, because of its efficient capability to separate EVs from contaminant proteins (especially from large initial volumes) UF-SEC gives a higher yield of pure vesicles if compared to those isolated by simple ultracentrifugation.

Interest on EVs is growing very fast over the years. Thanks to their characteristics (specifically their non-immunogenic nature due to the similar composition to the cell from which they originate) they were recently taken into consideration for their use as drug delivery vehicles (Batrakova and Kim, 2015).

Actually, liposomes are used as drug delivery vehicles, but their biocompatibility and their safety are still unknown. Unlike these synthetic systems, exosomes have long circulating half-life, promise to be biocompatible and stable, and have minimal or no inherent toxicity issues. Moreover, thanks to their small size, they are able to cross the blood-brain barrier (BBB), thus providing a useful carrier for the delivery of small drugs across this area.

Ninety-eight percent of drugs potentially important for the central nervous system cannot cross the BBB and their conceptual efficacy shown in labs has not a counterpart in clinical trials (Haney et al., 2015). Moreover, thanks to their capability to carry different molecules (protein and miRNA among others), they can also eliminate problems related to the instability of nucleic acid based drugs (Liang et al., 2018). Furthermore, the possibility to isolate them from all biological fluids, suggests their use for diagnostic applications, providing a non-invasive diagnostic method. For instance, they can be used for diagnosis since circulating exosomes can be correlated to specific diseases (Aryani and Denecke, 2016). For these reasons, if compared with their synthetic counterparts, they seem to be a better choice, exceeding those synthetic nanoparticles limitations. That’s why they have aroused a lot of interest as a drug delivery system for the treatment of several chronic and neurodegenerative diseases (Ha et al., 2016).

Nowadays their use as a drug delivery system for small molecules, proteins and nucleic acids is already a true reality; Alvarez-Erviti et al. (2011) showed that intravenously injected Rabies Virus Glycoprotein (RVG) targeted exosomes delivered GAPDH siRNA specifically in the brain, resulting in a specific gene knockdown. A similar result was also confirmed by Liu et al. (2015), showing that exogenous siRNA transfected into cells can be packaged by exosomes and delivered into recipient cells to regulate gene silencing, indicating that exosomes can serve as siRNA delivery vesicles in gene therapy for cancer and other diseases. In another study, large size plasmid DNA encapsulated into exosomes was successfully transferred to MSCs (Lin et al., 2018). Sun et al. (2010) treated mice with curcumin carrying exosomes, demonstrating that they were protected against lipopolysaccharide (LPS)-induced septic shock. Curcumin was more stable both *in vitro* and *in vivo*. In a similar study, Gomari et al. (2018) encapsulated doxorubicin (a drug currently used for breast and solid cancer) into exosomes to increase local dosage of the molecule and reduce its adverse effects on other organs. Considering their ability to deliver large molecules (such as proteins), Khatua et al. (2009) demonstrated that human cytidine deaminase APOBEC3G (A3G), a cellular defense system against human immunodeficiency virus type 1 (HIV-1) and other retroviruses, can be secreted in exosomes conferring an antiviral phenotype to target cells, also limiting replication of the virus in recipient cells.

However, despite several evidences of their potential as drug delivery system, one of the principal obstacles for the application of exosomes in clinic is their final yield from donor cells, which is often very limited and strongly related to the protocol of isolation (Van Deun et al., 2014). In addition, they may act as vehicles for the replication and propagation of transmissible pathogens, since exosomes derived from bacteria, or virus-infected cells, might contain pathogen-derived factors that activate a pro-inflammatory pathway. Plus exosomes have also a different effect on health and diseases; indeed, despite some of them can prevent tumor development (Naseri et al., 2018; Rosenberger et al., 2019), others provide a communication system between tumor cells and the surrounding tissues (Haga et al., 2015; Keklikoglou et al., 2019).

In the last few years, researchers have started to combine exosomes with synthetic nanoparticles, developing engineered particles more efficiently than their natural counterparts do. For instance, Sato et al. (2016), to control and modify the performance of exosomal nanocarriers, realized hybrid exosomes fusing them with polyethylene glycol (PEG) liposomes. This modification facilitated cellular uptake of the PEG modified exosomes, reducing also their circulation time in the blood. Other researchers created exosome-mimetic nanovesicles by serial extrusions through polycarbonate membranes with pore sizes of 10, 5, and 1 μm, for their utilization in tissue repair and regeneration. These exosome-mimetic nanovesicles (NVs) had a final yield almost 100 times higher than exosomes and promoted cell proliferation and liver regeneration similar to that induced by exosomes (Wu et al., 2018).

## EVs and Exercise: EVs Changes Induced by Exercise

The capacity of myokines to positively influence the metabolism and homeostasis of the body, makes them promising targets for treatment of several diseases. However, little is known about the mechanisms that regulate the release of these factors, especially regarding the final steps in recruitment and exocytosis of specific secretory vesicles. In fact, despite muscle cells express several secretory vesicle transport proteins (Romancino et al., 2013), the mechanisms that target containing vesicles to particular regions in the plasma membrane to control myokine secretion are largely unknown. Some studies demonstrated that glucose receptor GLUT4 is translocated to the plasma membrane in Vesicle-Associated Membrane Protein 2 (VAMP2) labeled vesicles and, its translocation, requires an active remodeling of actin filament which can be induced by insulin (Giudice and Taylor, 2017).

Exercise triggers the release of exerkines (EXs) into the circulation, possibly through their encapsulation within EVs (Gorgens et al., 2015; Lombardi et al., 2016) that mediates the systemic benefits of physical exercise, in both physiological and pathological conditions (Safdar et al., 2016). In fact, it was shown that in patients with cardio-metabolic risk factors, acute exercise promoted a large release of plasma EVs (Bei et al., 2017). A similar mechanism of communication among cells was already seen during high energy demand related to exercise; in this case, cells released enzymes of the glycolytic pathway into the EVs, that probably influenced the glycolytic rate in the recipient cells (Garcia et al., 2016; Zhao et al., 2016).

Whitham et al. (2018) analyzed (by means of the Nano-UHPLC followed by mass spectrometry) the proteome of the EVs of human plasma (before and after exercise), demonstrating how the EV trafficking was involved in tissue cross talk during physical activity. Indeed, exercise induces an increase of more than 300 proteins in the circulation, many of them associated with the biogenesis and function of “small vesicles” and exosomes. They identified 35 new myokine candidates, supporting the idea that the skeletal muscle is one of the major distributors of secreted molecules during exercise (Deshmukh et al., 2015). Some of these molecules were also found in EVs collected from myotubes conditioned medium (Forterre et al., 2014), but also from plasma and serum of participants who had walking speed decline (Suire et al., 2017). Another interesting data obtained by Petersen et al. (2011), showed the amino acid sequences of each protein transported inside these vesicles. Using the SignalP 4.0 server, they revealed that these proteins were deprived of the signal peptide sequence, typical of the classical secretion pathway. According to the obtained results, they postulated that the increase of EVs into the circulation (induced by exercise), can be related to the mechanism by which the skeletal muscle releases myokines, in a way independent of the classic secretory pathway. Moreover, exosome release is generally associated with an increase of intracellular calcium (Savina et al., 2003); since the motoneuron stimulates skeletal muscle fibers (causing an immediate release of Ca^2+^ from the sarcoplasmic reticulum) (Melzer et al., 1984), it may be plausible that the release of muscle small vesicles is faster than in other organs. However, uptake of the EVs in the recipient cells during exercise is a necessary step to talk about tissue cross talk and the reduction of the amount of proteins within the EVs (4 h after exercise), suggests that these proteins are partially removed from the circulation by tissue absorbing.

Therefore, exosomes and sEVs may act as communicator factors among cells, through the packaging of proteins inside their lumen.

## Conclusion

Even though it has been widely proved that, during exercise, muscle cells release several myokines into the circulation with a potential role in whole-body homeostasis (Aswad et al., 2014), the mechanisms underlying this secretion process are still vaguely known. Since calcium is an essential ion involved in both extracellular vesicles secretion and contraction of skeletal muscle fibers, it may be possible that, during exercise, the stimulation of muscle cells from motoneurons enhances the release of small vesicles potentially carrying myokines. However, there are almost no studies about that, hence, further analysis are needed in order to better understand the relationship between nanovesicles and myokines.

## Author Contributions

All authors listed have made a substantial, direct and intellectual contribution to the work, and approved it for publication.

## Conflict of Interest Statement

The authors declare that the research was conducted in the absence of any commercial or financial relationships that could be construed as a potential conflict of interest.
